# Technique and Preliminary Results of Sole-echocardiography-guided Tricuspid Transcatheter Edge-to-edge Repair without the Use of Fluoroscopy

**DOI:** 10.31083/j.rcm2511413

**Published:** 2024-11-21

**Authors:** Manchen Gao, Hao Shi, Cheng Wang, Hong Meng, Jiande Wang, Da Zhu, Zhiling Luo, Chuangshi Wang, Ziping Li, Junke Chang, Fengwen Zhang, Xiangbin Pan, Shouzheng Wang

**Affiliations:** ^1^Department of Structural Heart Disease, National Center for Cardiovascular Disease, China & Fuwai Hospital, Chinese Academy of Medical Sciences & Peking Union Medical College, 100037 Beijing, China; ^2^National Health Commission Key Laboratory of Cardiovascular Regeneration Medicine, 100037 Beijing, China; ^3^State Key Laboratory of Cardiovascular Disease, 100037 Beijing, China; ^4^Key Laboratory of Innovative Cardiovascular Devices, Chinese Academy of Medical Sciences, 100037 Beijing, China; ^5^National Clinical Research Center for Cardiovascular Diseases, Fuwai Hospital, Chinese Academy of Medical Sciences, 100037 Beijing, China; ^6^Department of Echocardiography Imaging Center, National Center for Cardiovascular Diseases, China & Fuwai Hospital, Chinese Academy of Medical Sciences & Peking Union Medical College, 100037 Beijing, China; ^7^Department of Structural Heart Disease, Fuwai Yunnan Cardiovascular Hospital, 650102 Kunming, Yunnan, China; ^8^Department of Ultrasonography, Fuwai Yunnan Cardiovascular Hospital, 650102 Kunming, Yunnan, China

**Keywords:** tricuspid regurgitation, tricuspid transcatheter edge-to-edge repair, echocardiography, sole-echocardiography-guided

## Abstract

**Background:**

Tricuspid transcatheter edge-to-edge repair (T-TEER) has emerged as an attractive option for severe tricuspid regurgitation (TR). To avoid the radiation exposure for both patients and medical staff, we propose a sole-echocardiography-guided method for T-TEER. The purpose of this article was to investigate the feasibility of sole-echocardiography-guided T-TEER.

**Methods:**

This was a retrospective observational study, including 43 patients who underwent sole-echocardiography-guided T-TEER at two medical centers in China between June 2022 and September 2023. Clinical and echocardiographic data were collected at baseline, discharge and 6-month follow-up.

**Results:**

Patients enrolled in this study were elderly (71.6 ± 8.2 years) with significant comorbidities, 67.4% had baseline massive or torrential TR and 76.7% were classified as New York Heart Association (NYHA) functional class III/IV. All patients achieved successful device implantation, and no severe device-related complications or mortality occurred during the follow-up period. Significantly reduced TR and reversed right ventricular remodeling were observed at 6-month follow-ups. Patients classified as NYHA functional class I/II increased from 23.3% at baseline to 81.4% at 6-month follow-up (*p* < 0.001).

**Conclusions:**

Sole-echocardiography-guided T-TEER has a low incidence of complications and can effectively reduce TR. It is feasible to substitute conventional fluoroscopy and echocardiography guidance for echocardiography guidance alone. Further large-scale randomized controlled trials are needed to validate the safety, efficacy and patient benefits of this technique.

## 1. Introduction

Tricuspid regurgitation (TR) is a highly prevalent valvular heart disease which can lead to right-sided heart failure, edema, liver congestion and renal injury, and is associated with poor long-term outcomes. While drug therapy can improve the symptoms of patients, it does not affect the long-term prognosis of TR [1]. Surgical management is recommended for severe TR only when left-sided heart valve surgery is performed [2]. The perioperative mortality of isolated tricuspid valve surgery can be as high as 8% to 10% [3, 4].

Over the past 5 years, transcatheter intervention of the tricuspid valve has emerged as an alternative strategy, which includes transcatheter tricuspid valve replacement, annuloplasty, and transcatheter edge-to-edge repair (TEER). Given the successful experience of TEER in management of mitral regurgitation, tricuspid transcatheter edge-to-edge repair (T-TEER) is now considered a promising option for severe or greater TR. Several international clinical studies have demonstrated the safety and effectiveness of T-TEER [5, 6, 7, 8]. T-TEER has been approved by both the Food and Drug Administration (FDA) of America and European Conformity (known as CE) of Europe. The TriClip (Abbott, Santa Clara, CA, USA) and PASCAL (Edwards Lifesciences, Irvine, CA, USA) implant systems have been used in T-TEER.

The conventional TEER procedure requires both fluoroscopy and echocardiography guidance. To avoid the radiation exposure for both patients and medical staffs, our team has developed a percutaneous and non-fluoroscopic procedure. This procedure relies exclusively on echocardiographic guidance without any fluoroscopy exposure, and has been applied in various treatments of structural heart disease, such as mitral valve TEER, percutaneous balloon mitral valvuloplasty, and atrial defect occlusion [9, 10, 11]. Compared with sole-echocardiography-guided mitral valve TEER, T-TEER has a closer anatomical relationship with the inferior vena cava and does not require atrial septal puncture. Consequently, the procedure for sole-echocardiography-guided T-TEER is easier. In this study, we present the results of sole-echocardiography-guided T-TEER.

## 2. Methods

### 2.1 Study Design and Patient Cohort

This retrospective, observational, cohort study was conducted at Fuwai Hospital, Chinese Academy of Medical Sciences & Peking Union Medical College, and Fuwai Yunnan Cardiovascular Hospital. The study received approval from the ethics committee of each hospital (approval numbers: 2022-1853 and 2022-005-01). All patients and legal guardians signed an informed consent for the operation and clinical record review.

A total of 43 consecutive patients who underwent sole-echocardiography-guided T-TEER from June 2022 to September 2023 were enrolled, no exclusion criteria were defined. 12 patients were enrolled from Fuwai Hospital and 31 patients were enrolled from Fuwai Yunnan Cardiovascular Hospital.

### 2.2 Echocardiographic Assessment

All patients underwent transthoracic or transesophageal echocardiography (TEE) examinations before operation, at discharge and at 6-month follow-up. TR was graded using a pre-specified 5-class grading scheme: mild, moderate, severe, massive, and torrential [12]. Echocardiographic data included quantitative indicators of TR, such as effective regurgitation area, regurgitation volume and vena contracta. Right-sided heart chamber size and functional indicators were also documented.

### 2.3 Procedure 

All T-TEER procedures were performed in a hybrid operation room with standby Digital Subtraction Angiography (DSA). The TEER devices used in this study were the Kyrin Transcatheter Tricuspid Valve Repair System (Shenqi Medical, Shanghai, China) and the Neoblazar Transcatheter Tricuspid Valve Clipping Device and Delivery System (Trulive MedTech, Jiangsu, China). The procedure described in this paper is the Kyrin system.

**Fig. 1.**
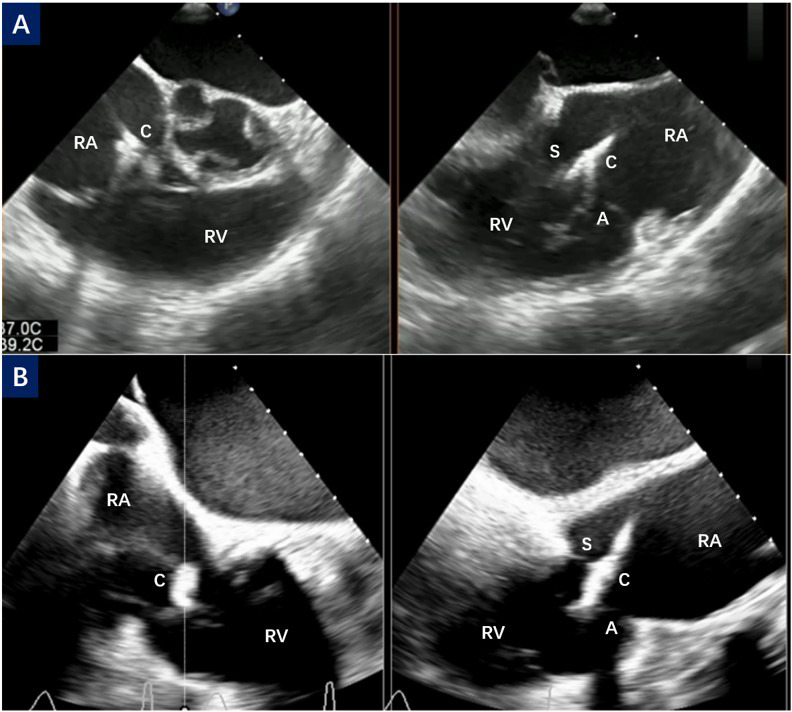
**CDS orientation and septal hugger**. (A) The right ventricular inflow/outflow tract view and biplane view of TEE show the orientation process of CDS toward the tricuspid valve. (B) The CDS points obliquely to the tricuspid valve, also known as the septal hugger. C, clip; S, septal leaflet; A, anterior leaflet; RA, right atrium; RV, right ventricular; TEE, transesophageal echocardiography; CDS, Clip Delivery System.

The positional relationship between the guide catheter and the Clip Delivery System (CDS) was pre-measured and marked on CDS. Under the guidance from a bicaval view of the TEE, the head of a steerable guide catheter was straightened and directed along the guide wire to the middle of the right atrium. Then, the CDS was delivered from the guide catheter to the marked depth to complete the alignment of the catheter and the CDS. The TEE showed the right ventricular inflow/outflow tract view and biplane view, with rotation of the Flex/Extend (F/E) knob on the CDS in the F direction to orientate it towards the tricuspid valve (Fig. 1). When the CDS is tilted from the septum toward the tricuspid valve (Septal Hugger, Fig. 1), it can be adjusted by rotating the Septal/Lateral (S/L) knob in the L direction and rotating the guide catheter clockwise.

**Fig. 2.**
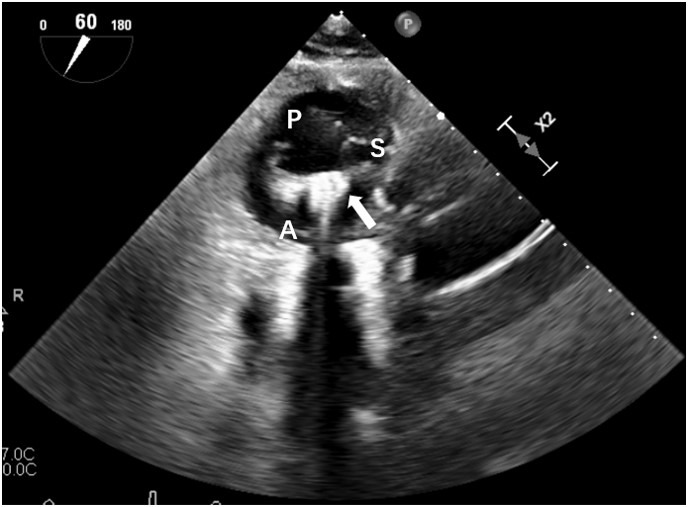
**Orientation of clip arm**. The deep transgastric short-axis view of TEE shows the orientation process of clip arm perpendicularly to the coaptation margin between the septal leaflet and the anterior leaflet. P, posterior leaflet; S, septal leaflet; A, anterior leaflet; Arrow, clip with opening arm; TEE, transesophageal echocardiography.

**Fig. 3.**
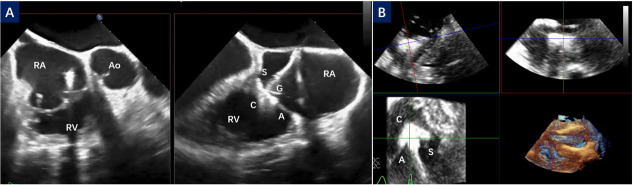
**Leaflet capture in different TEE view**. (A) The right ventricular inflow/outflow tract view and biplane view; (B) Multiview of the deep transgastric short-axis image. C, clip; G, gripper; S, septal leaflet; A, anterior leaflet; RA, right atrium; RV, right ventricular; Ao, aorta; TEE, transesophageal echocardiography.

The subsequent steps are similar to the traditional fluoroscopy and TEE guidance. The key steps are illustrated in Figs. 2,3.

After capturing the leaflet and closing the clip, leaflet insertion and grasping, the residual TR and tricuspid valve gradient assessment were performed. The Clip was released in a standard fashion. Deployment of more than one Clip device was allowed if necessary.

### 2.4 Clinical Data and Follow-up

Baseline patient demographics and in-hospital data were obtained from the medical history system of each hospital. Transthoracic echocardiographic follow-up was routinely performed at discharge and 6 months post procedure.

### 2.5 Statistical Analysis

Statistical analysis was performed using SPSS Statistics 26.0 (SPSS Inc., Chicago, IL, USA). A *p*-value of less than 0.05 was considered statistically significant. Continuous variables were presented as mean ± standard deviation, while categorial variables were specified in absolute numbers and percentages. A paired Student’s *t*-test was utilized to compare specific time points with baseline for continuous variables, while the McNemar’s test was employed to compare paired categorical variables.

## 3. Results

### 3.1 Baseline Characteristics

A total of 43 patients were enrolled in this study, including 21 males and 22 females, with a mean age of 71.6 ± 8.2 years. The most common comorbidities were atrial fibrillation (32, 74.4%), hypertension (18, 41.9%), coronary artery disease (14, 32.6%), arrhythmia other than atrial fibrillation (14, 32.6%), hyperuricemia (13, 30.2%) and chronic obstructive pulmonary disease (13, 30.2%). 6 patients (14.0%) had previous cardiac surgery (including aortic valve replacement, mitral valve replacement, atrial septal defect repair and thoracoscopic radiofrequency ablation of atrial fibrillation), and 9 (20.9%) had previous percutaneous cardiac interventions (including percutaneous coronary intervention, radiofrequency catheter ablation and permanent pacemaker implantation). 29 (67.4%) patients had baseline massive or torrential TR and 33 (76.7%) were classified as New York Heart Association (NYHA) functional class III/IV. Baseline characteristics are summarized in Table 1.

**Table 1.** **Baseline characteristics**.

Variable		All Subjects (n = 43)
Age (years)		71.6 ± 8.2
Female (%)		22 (51.2%)
Atrial Fibrillation		32 (74.4%)
Hypertension		18 (41.9%)
Coronary Artery Disease		14 (32.6%)
Arrhythmia other than Atrial Fibrillation		14 (32.6%)
Hyperuricemia		13 (30.2%)
Chronic Obstructive Pulmonary Disease		13 (30.2%)
Renal Disease		8 (18.6%)
Diabetes		8 (18.6%)
Cardiac Intervention-Surgery		6 (14.0%)
	Prior Aortic Replacement	2 (4.7%)
	Prior Mitral Replacement	3 (7.0%)
Cardiac Intervention-Percutaneous		9 (20.9%)
	Percutaneous Coronary Intervention	4 (9.3%)
	Radiofrequency Catheter Ablation	3 (7.0%)
	Permanent Pacemaker	2 (4.7%)
Secondary TR		42 (97.7%)
	Atrial Secondary TR	20 (46.5%)
	Ventricular Secondary TR	22 (51.2%)
Primary TR		1 (2.3%)
NYHA FC III/IV		33 (76.7%)
LVEF (%)		59.02 ± 9.27
6MWD (m)		297.58 ± 97.80 (n = 26)
NT-proBNP (pg/mL)		1409.34 ± 1487.50

TR, tricuspid regurgitation; NYHA FC, New York Heart Association functional class; LVEF, left ventricular ejection fractions; 6MWD, 6-min walk distance; NT-proBNP, N-terminal pro-B-type natriuretic peptide.

### 3.2 Procedural Outcomes

**Fig. 4.**
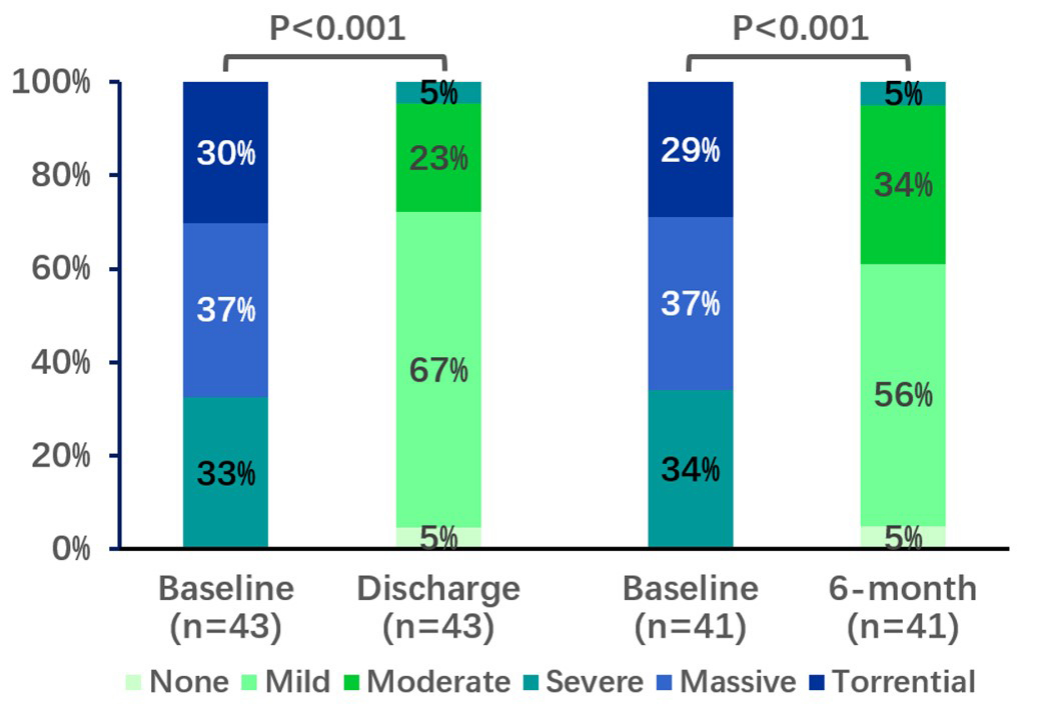
**Change in tricuspid regurgitation grade up to 6-month follow-up**. *p* values were calculated by McNemar’s test for paired variables.

21 patients underwent tricuspid TEER procedure with the Kyrin System and another 22 patients had the Neoblazar System. Successful implantation was achieved in all patients. An average of 1.9 ± 0.68 clips were deployed, and the mean device time was 77.5 ± 39.03 minutes. 12 patients were implanted with one clip, 23 patients with two clips and 8 patients with 3 clips. Type WL clips (6 mm width and 9 mm length, or the same parameters) were used for all devices except one NL clip (4 mm width and 9 mm length). 41 patients were clipped between the septal and anterior leaflet, while 2 other patients were clipped at both the septal-anterior leaflet and septal-posterior leaflet. All cases achieved a minimum TR reduction of one grade and 95% of the patients had TR no greater than moderate at discharge (Fig. 4). Only 2 patients exhibited severe TR upon discharge, both had preoperative torrential TR. No intraoperative or postoperative device-related complications such as single leaflet device attachment (SLDA) occurred. One patient developed a femoral arteriovenous fistula after T-TEER, and the fistula disappeared after 2 days of compression and immobilization.

### 3.3 Follow-up

Follow up was completed for all patients, with a median of 189 (164–377) days. 41 patients completed the outpatient visit and TTE examination at 6 months after surgery. Another 2 patients were unable to attend the follow-up examinations at the research hospital due to personal circumstances, so telephone interviews were conducted as an alternative. As a result, ultrasound data for these two patients could not be obtained.

No mortality was observed during the follow-up period. A total of 12 patients had re-hospitalizations. Among them, 4 patients had 5 re-hospitalizations for heart failure, and another four patients had re-hospitalizations for arrhythmias. Femoral vein thrombosis occurred in 2 patients, and their symptoms improved after receiving advanced anticoagulant therapy. There were no device-related complications (such as SLDA) and no tricuspid valve re-intervention or reoperation during follow-up.

The result was durable with 95% of patients having a sustained TR ≤2+ (Fig. 4). Compared to the discharge results, the proportion of TR 2+ increased and the proportion of TR 1+ decreased. At 6 months after operation, the mean transvalvular pressure gradient was 1.59 ± 0.88 mmHg (0.4–5 mmHg).

Significant reductions of TR were observed by echocardiography, including reductions of effective regurgitation orifice area (0.62 ± 0.19 to 0.16 ± 0.08 cm^2^, *p* < 0.001), regurgitant volume (56.65 ± 16.74 to 13.80 ± 9.00 mL/beat, *p* <0.001), vena contracta width (1.06 ± 0.27 to 0.38 ± 0.17 cm, *p* < 0.001), proximal isovelocity surface area (PISA) radius (0.85 ± 0.17 to 0.28 ± 0.12 cm, *p* < 0.001) and diameter of the inferior vena cava (2.07 ± 0.47 to 1.75 ± 0.42 cm, *p* < 0.001) between baseline and 6-month follow-up. Right heart reverse remodeling was also documented, including a decrease of right ventricular end diastolic dimension (4.32 ± 0.55 to 3.91 ± 0.53 cm, *p* < 0.001), tricuspid annular diameter (4.20 ± 0.47 to 3.72 ± 0.43 cm, *p* < 0.001) and right atrium volume (105.95 ± 33.46 to 88.39 ± 20.47 mL, *p* < 0.001). No significant changes were observed in right ventricular (RV) function parameters, such as RV fractional area change (FAC) or tricuspid annular plane systolic excursion (TAPSE). The data is summarized in Table 2.

**Table 2.** **Summary of echocardiographic data**.

Echocardiographic data		Baseline	Discharge	6-month	*p*-value Baseline vs 6-month
Tricuspid Regurgitation					
	Effective Regurgitation Orifice Area, cm^2^	0.62 ± 0.19	0.15 ± 0.08	0.16 ± 0.08	<0.001
	Regurgitant Volume, mL/beat	56.65 ± 16.74	10.88 ± 6.79	13.80 ± 9.00	<0.001
	Vena Contracta Width, cm	1.06 ± 0.27	0.30 ± 0.15	0.38 ± 0.17	<0.001
	PISA Radius, cm	0.85 ± 0.17	0.23 ± 0.09	0.28 ± 0.12	<0.001
	IVC Diameter, cm	2.07 ± 0.47	1.80 ± 0.42	1.75 ± 0.42	<0.001
Right Heart Reverse Remodeling					
	RV End Diastolic Dimension, cm	4.32 ± 0.55	4.00 ± 0.48	3.91 ± 0.53	<0.001
	Tricuspid Annular Diameter, cm	4.20 ± 0.47	3.88 ± 0.41	3.72 ± 0.43	<0.001
	Right Atrial Volume, mL	105.95 ± 33.46	89.60 ± 25.99	88.39 ± 20.47	<0.001
	RV Fractional Area Change, %	39.36 ± 10.35	37.86 ± 8.31	39.68 ± 7.20	0.895
	TAPSE, cm	1.73 ± 0.39	1.70 ± 0.34	1.73 ± 0.32	0.938

PISA, proximal isovelocity surface area; IVC, inferior vena cava; RV, right ventricular; TAPSE, tricuspid annular plane systolic excursion.

**Fig. 5.**
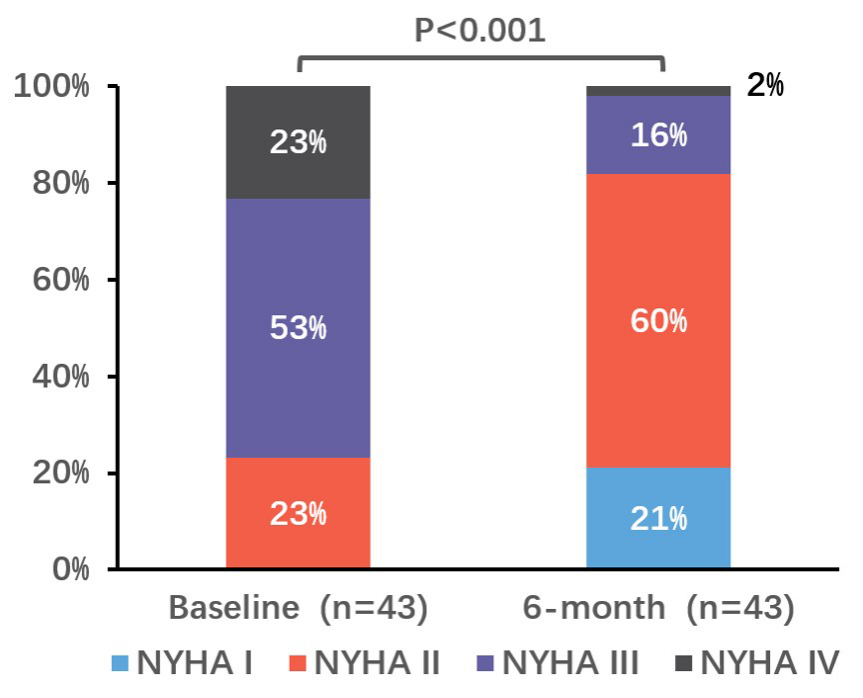
**Change in NYHA functional class at 6 months**. *p* values were calculated by McNemar’s test for paired variables. NYHA, New York Heart Association.

Significant improvements in functional capacity were observed at 6-month follow-up. The proportion of subjects classified as NYHA functional class I/II increased from 23% at baseline to 81% at 6-month follow-up (*p* < 0.001, Fig. 5). A total of 28 patients completed the 6-minute walk distance (6MWD) examination at 6-months with a mean distance of 356.79 ± 89.54 meters. 18 patients completed both the preoperative and postoperative 6MWD assessments, and a paired *t*-test revealed a significant increase from preoperative to postoperative results (297.58 ± 97.80 meters vs 376.17 ± 105.74 meters, *p* = 0.002).

## 4. Discussion

In this study, we present the technique of sole-echocardiography-guided T-TEER and the preliminary results in a real-world cohort of patients with symptomatic TR. All patients achieved successful device implantation with a low incidence of adverse events. After a 6-month follow-up, patients exhibited reduced TR, decreased right heart size and improved cardiac function. These results were consistent with previous studies, confirming the safety, durability and effectiveness of T-TEER in TR patients, and illustrated the feasibility of this approach.

In this study, our cohort exhibited similar baseline characteristics to other international registries, such as the TRILUMINATE trial and the bRIGHT registry, with comparable comorbidities, 67% of patients presented with massive and torrential TR and 76.7% were in NYHA class III/IV [6, 7, 8]. Our cohort had similar TR severity but smaller right heart size. This observation may be attributed to the relatively lower body weight commonly observed in Asian populations compared to European and American populations. Specifically, in Yunnan Province, China, both height and body weight of the population are below average for the Chinese population. Our results reveal a reduced incidence of mortality and device-related complications, potentially attributed to the limited sample size in our study and the expertise of the participating surgeons. During the follow-up period, we observed statistically significant differences in the reduction of TR severity and a decrease in right heart size, consistent with findings from other studies. Although there was an initial decline indicator of right ventricular function, they subsequently returned to preoperative levels at the 6-month follow-up, consistent with the findings of the BRIGHT study in Europe [8]. We believe that right heart functional indicators may show differences after extended pharmacological treatment and long-term follow-up.

Radiation exposure is a persistent problem in interventions for structural heart disease. It has been reported that the median radiation dose per case in echocardiographers and interventional cardiologists in mitral valve TEER was 10.6 µSv and 0.9 µSv, respectively [13]. With the development of new devices and advancements in imaging techniques, intraoperative echocardiographic guidance is likely to become increasingly important in transcatheter structural cardiac interventions [14]. Therefore, we present these findings of the T-TEER procedure guided solely by echocardiography. This procedure offers numerous advantages, including the reduction of radiation exposure for both patients and medical staff, independence from bulky DSA equipment, and reductions in medical costs. This procedure requires a comprehensive interpretation of ultrasound images and entails a certain learning curve. Therefore, it is necessary to validate the safety of this procedure through large-scale multicenter clinical trials. We believe that the sole-echocardiography-guided procedure holds immense potential and it will gain significant attention in the future.

In the routine procedure for T-TEER, radiation guidance is used in the process of inserting the guiding sheath, aligning the guiding sheath and CDS, and orienting it toward the tricuspid valve. In contrast, when solely relying on TEE guidance, the bicaval view provides visualization of key anatomical structures such as the inferior vena cava, superior vena cava, and right atrium. This facilitates clear observation of the wire entry into the superior vena cava and sheath insertion into the right atrium for precise positioning of its distal end. During CDS insertion, external measurements are utilized to determine its depth within the sheath for accurate alignment. Simultaneously, the TEE bicaval view offers a distinct display of the clip head position in the right atrium to prevent damage to the atrium. The combination of the TEE bicaval view and right ventricular inflow/outflow tract view allows real-time monitoring of the CDS orientation towards the tricuspid valve.

Due to the anterior location and distance from the esophagus, the tricuspid valve image on the TEE is susceptible to interference from left-sided heart prosthetic valves and CDS. The mid or lower esophageal short-axis view may not provide sufficient clarity for visualizing clip capture of the tricuspid valve. By utilizing a 20°–60° transgastric short-axis view, we can avoid artifacts from the left heart or delivery system while simultaneously displaying all three leaflets of the tricuspid valve in a two-dimensional plane. Using Biplane functionality on this view allows us to visualize both the anterior and posterior leaflets of the tricuspid valve on right ventricular long-axis images, enabling assessment of clip insertion depth into the right ventricle. Utilizing gastric short-axis images for three-dimensional reconstruction and employing multiview for image segmentation reconstruction enables visualization of the relationship between the clip and the leaflets, guiding leaflet capture. Therefore, the transgastric short-axis view plays a crucial role in these important steps such as adjusting the clamp position and orientation, guiding leaflet capture, and assessing the degree of leaflet grasping during the T-TEER procedure [15].

The sole-echocardiography-guided method may also present some limitations. Echocardiographic images may not be as clear as radiographic images during some steps, such as aligning the guide catheter and CDS, and orientating the CDS towards the tricuspid valve. In these steps, the cardiologist needs to carefully manipulate the system and follow the methods described above. Moreover, for patients with poor acoustic windows (e.g., severe right heart enlargement, cardiac rotation, and previous implantation of cardiac prostheses), the use of sole echocardiographic guidance becomes more challenging. In such cases, intracardiac echocardiography (ICE) can offer supplementary imaging for procedural guidance [16].

## 5. Limitations

Due to the retrospective nature of this study, some important data were missing or incomplete during the follow-up period. For instance, there was a lack of assessment the patients’ quality of life with missing Kansas City cardiomyopathy questionnaire (KCCQ) scores. 6MWD data was incomplete and only 18 cases had paired data available. Other limitations of this study include the absence of a control group, small sample size, and short follow-up period. Further large-scale clinical randomized controlled trials are needed to validate the safety, efficacy and patient benefits of sole-echocardiography-guided T-TEER.

## 6. Conclusions

In this study, we present a novel technique for the sole-echocardiography-guided TEER procedure, which has resulted in a significant reduction in TR severity, improvements in right heart morphology, and a low incidence of complications during the 6-month follow-up period. These results are consistent with international studies on T-TEER and confirm the feasibility of our approach.

## Availability of Data and Materials

The data sets generated and analyzed during the current study are not publicly available due to regulation of Ethics Committee, but are available from the corresponding author on reasonable request.
